# The Golden Activity of *Lysinibacillus sphaericus:* New Insights on Gold Accumulation and Possible Nanoparticles Biosynthesis

**DOI:** 10.3390/ma11091587

**Published:** 2018-09-02

**Authors:** María Camila Bustos, Humberto Ibarra, Jenny Dussán

**Affiliations:** 1 Microbiological Research Center (CIMIC), Department of Biological Sciences, Universidad de los Andes, Bogotá 111711, Colombia; mc.bustos198@uniandes.edu.co; 2 Microscopy Center, Universidad de los Andes, Bogotá 111711, Colombia; hibarraavila@uniandes.edu.co

**Keywords:** mining, gold, *Lysinibacillus sphaericus*, sorption, nanoparticles, SEM

## Abstract

Power struggles surrounding the increasing economic development of gold mining give rise to severe environmental and social problems. Two new strains of *Lysinibacillus sphaericus* were isolated from an area of active alluvial gold mining exploitation at El Bagre, Antioquia. The absorption capacity of these strains and some of the *L. sphaericus* Microbiological Research Center (CIMIC) collection (CBAM5, OT4b.31, III(3)7) were evaluated by spectrophotometry according to a calibration gold curve of HAuCl_4_^−^ with concentrations between 0 µg/mL and 100 µg/mL. Bioassays with living biomass were carried out with an initial gold concentration of 60 µg/mL. Their sorption capacity was evident, reaching percentages of gold removal between 25% and 85% in the first 2 h and 75% to 95% after 48 h. Biosynthesis of possible gold nanoparticles (AuNPs) in assays with living biomass was also observed. Metal sorption was evaluated using scanning electron microscopy and energy-dispersive X-ray spectroscopy (EDS) analysis. The sorption and fabrication capacity exhibited by the evaluated strains of *L. sphaericus* converts this microorganism into a potential alternative for biomining processes, especially those related to gold extraction.

## 1. Introduction

Mining is an important worldwide activity; indeed, it influences and involves economic, political, environmental, and social aspects in a complex matrix. The exploitation of metallic and non-metallic minerals can be carried out in soil, sub-soil, or even in riverbeds. Nowadays, gold mining is a controversial topic; it is associated with negative impacts such as the adverse effects in terms of environmental pollution and high health risk due to excessive exposure to mercury and cyanide, especially in developing countries where illegal and artisanal mining are present [1].

In Colombia, the gold industry plays an important role in the economic development of the country. Colombia is the fourth producer of gold in South America, and is among the 20 major producers in the global ranking, besides developed countries and world powers as China, the United States, Russia, and Australia [2]. Because gold is the only financial asset which is not controlled, it is a meaningful commodity not only for Colombia but worldwide. Unfortunately, this is also the reason why the gold mining industry is associated with violence, power struggles in the country, and an excessive presence of illegal mining [3]. In Colombia, the highest percentage of environmental licenses and, consequently, exploited hectares are in the departments of Choco and Antioquia [2]. The direct repercussions in these areas are the adverse environmental effect on water bodies, destruction of the diversity, and important health problems emerging in the surrounding communities [4,5].

It is true that areas with active mine exploitation represent challenging environments for microorganisms, due to their extreme conditions such as high pressure and temperature, elevated salt concentrations, a diverse range of acid and alkaline soil/water, and many others abiotic factors [6]. However, an impressive diversity of microorganisms has been found in these types of habitats including those associated with gold mining activity, where communities of Phylum Proteobacteria, Firmicutes and Actinobacteria, are predominant [7,8,9,10]. Despite the fact that the most abundant organisms are bacteria, representatives of the Archea and Eukarya domains have also been found [11,12,13]. The ample metabolic capacity of the organisms present in these areas is evident, and their ability to act as Fe, S, NH_3_, and CH_4_ oxidants and SO_4_^2−^ reducers has been proven [8,14,15,16,17].

Microorganisms present great potential in sustainable mining, due to their metabolic capacities. For example, it has been demonstrated that some of them accelerate the process of sulfur oxidation and, as such, can be used in biomining and bioleaching [18]. Additionally, acidophilic microorganisms have allowed the development of different strategies for the remediation of important contamination problems in the exploitation of minerals; for example, the regulation and management of pH in the precipitation of iron in acid mine drainage (AMD) [19]. Furthermore, microorganisms, especially bacteria, have great potential in terms of the immobilization and accumulation of heavy metals such as Cu, Pb, Cr, and Fe, among others [20,21,22,23,24]. In this sense, they have the capacity to be used as an ideal alternative for remediation of contaminated sites, and an alternative to recover precious metals.

Gold exploitation is becoming increasingly important due to growing demand for the metal. This puts pressure on the industry to incorporate new methodologies and processes for efficient, less expensive, and ecologically sustainable extraction. In the biochemical cycle of the metal, secondary gold can be formed as a result of oxidation, solubilization, reduction, and precipitation of the ore under surface conditions due to microorganism activity [25]. Wide spectrums of organisms such as fungi, algae, and, in particular, bacteria have proved their potential for gold biomining at different stages of metal extraction and purification [26,27,28,29].

*Lysinibacillus sphaericus*, a sporulated gram-positive bacillus has become a focus of sustainable economic industry development because of its capacity for metal binding and remediation of contaminated matrices [30,31]. The first strain that exhibited metal binding capabilities was the JG-A12 (isolated from a uranium mining waste pile in Germany), which accumulated U, Cu, Pb, Al and Cd on its surface [32]. Others strains of the genus also manifest these sorption abilities with diverse metals such as Fe, Cr, Zn, among others [21,22,33].

The aim of the present study was to elucidate the ability of gold absorption by two new strains of *L. sphaericus* isolated from an area of active alluvial gold mining exploitation at El Bagre, Antioquia. The bioassays carried out showed efficiency in bioaccumulation and biosorption of gold in living cells, as well as the possible fabrication of nanoparticles.

## 2. Materials and Methods

### 2.1. Study Samples

Sampling was carried out in the exploitation territory of the company Mineros S.A., which specializes in alluvial gold mining in the Nechi River in the department of Antioquia, Colombia. This area is about 43,000 hectares with the following coordinates: 7°39′73 N, 74°47′22.73 O. Water samples were collected from different points throughout the gold exploitation process: Near to suction dredge, scoop dredge, moisture, and site of restoration at the initial phase. These samples were subjected to thermal shock to detect sporulated gram-positive bacilli. Five mL per sample was taken to be subjected to a temperature of 90 °C for 20 min. Dilutions from 10^−1^ and 10^−2^ were used. All cultures were carried out in duplicate and incubated for 48 h at 30 °C in nutrient agar (NA) [34].

### 2.2. L. sphaericus Strain Identification

For morphotypes identification, 16S rDNA gene amplification, sequencing, and phylogenetic analysis were carried out. Primers 27F (AGAGTTTGATCMTGGCTCAG) and 1493R (TACGGYTACCTTGTTACGACTT) [35] were used to amplify the 16S rDNA gene. For amplifications, 25-μL reactions were prepared containing 100 μM each dNTPs, 0.2 μM of each primer, 3 mM MgCl2, 2U Taq polymerase (Bioline), 1X PCR buffer, and 1 μL of crude extract from an overnight culture as the template DNA source. The amplification program consisted of a denaturing step of 94 °C for 3 min, 25 cycles of denaturing for 45 s at 94 °C, annealing for 45 s at 50 °C, and extension at 72 °C for 45 s and a final extension of 72 °C for 7 min. The PCR products were visualized in 1.0% agarose gels, then purified and sequenced by Macrogen Inc. (Seoul, Korea). Resulting sequences were compared with NCBI and RDP databases. The phylogenetic tree was constructed using sequences of *L. sphaericus* and *Bacillus* sp. reference strains obtained from GenBank. The phylogenetic tree choosen was the one that exhibited the minor Bayesian Information Criterion (BIC).

Additionally, characterization by the presence of S-layer protein and MTX and Bin toxins, and toxicology bioassays in larvae of *Culex quinquefasciatus* and *Aedes aegypti* were also performed [36]. The nucleotide sequences of the *L. sphaericus* strains from this study were deposited in the GenBank database under the accession numbers MH447519 and MH447518. A summary of the strains used in this study are shown in Table 1.

### 2.3. Calibration Curve for Gold Determination (HAuCl_4_)

For the calibration curve, 0.01 g of pure gold was dissolved in 20 mL of aqua regia and evaporated until the solution was near to dryness. Then, it was dissolved in approximately 2 mL of concentrated HCl and diluted with deionized water to 100 mL in a measuring flask [37]. Series of 20 mL for gold standard solutions between 0 and 100 µg/mL were used for the calibration curve. The absorption spectrum used was 313 nm and the linear regression equation obtained was A = 0.0251C—0.0101; R^2^ = 0.9996.

### 2.4. Metal Sorption in L. sphaericus Strains

Sorption was evaluated by spectrophotometry of the biomass suspended in minimal salt medium (MSM) with sodium acetate at 0.5% [21]. Experiments were carried out in triplicate and with a HAuCl_4_^−^ concentration of 60 µg/mL. The total volume was 15 mL in 50 mL flasks with a concentration between 10^6^ and 10^7^ UFC/mL. Gold within the cells was determined through supernatant measurements taken every 15 min for 2 h [21] and a final determination was made at 48 h for the possible formation of AuNPs.

The bioassays were carried out using CBAM5, OT4b.31 and III(3)7 strains, from the *L. sphaericus* CIMIC collection [30,38,39]. For these strains, the determinations were carried out using the mixture of the three strains and CBAM5 alone. The latter (CBAM5) was used for methods calibration due to its capacity to capture mercury [38]. This metal is highly likely to be found in the Nechi River, due to the presence of illegal and artisanal gold mining. Additionally, bioassays using the isolated strains of this study were also performed: *L. sphaericus* MCB1 and MCB2 as individual strains and as a mixture. The light, oxygen, temperature, and agitation conditions were controlled, keeping the experiments in darkness with 150 rpm and a constant temperature of 30 °C [26].

### 2.5. SEM and EDS Analysis

The samples were fixed in 2.5% glutaraldehyde for 7 h followed by a process of dehydration through rinsing in an increasing ethanol gradient [40]. Modifications of previous protocol were carried out for the final steps. For sample mounting, briefly, a drop of approximately 5 uL of each sample was placed on an aluminum support and dried at room temperature for its posterior SEM observation and metal semi quantitation with energy-dispersive X-ray spectroscopy (EDS) using a JEOL JSM-6490LV (JEOL, Tokyo, Japan) scanning electron microscope equipped with an Oxford INCA PentaFetX3 EDS detector.

Samples were prepared for metal determination inside the cell, using a modification of previous protocols [41]. After glutaraldehyde fixation, a gradient between ethanol and resin was carried out until the sample was 100% embedded in just resin. Then, the sample was heated for 24 h at 56 °C. After polymerization, samples were cut using Leica ULTRACUT UC7 microtome to obtain 70 nm thick slices, which were placed on 200 mesh cupper grids. Uranyl acetate was used as contrast agent. Samples were observed using a Tescan LYRA 3 Scanning electron microscope (TESCAN, Brno, Czech Republic).

## 3. Results

### 3.1. Sporulated Microorganisms

**Figure 1:**
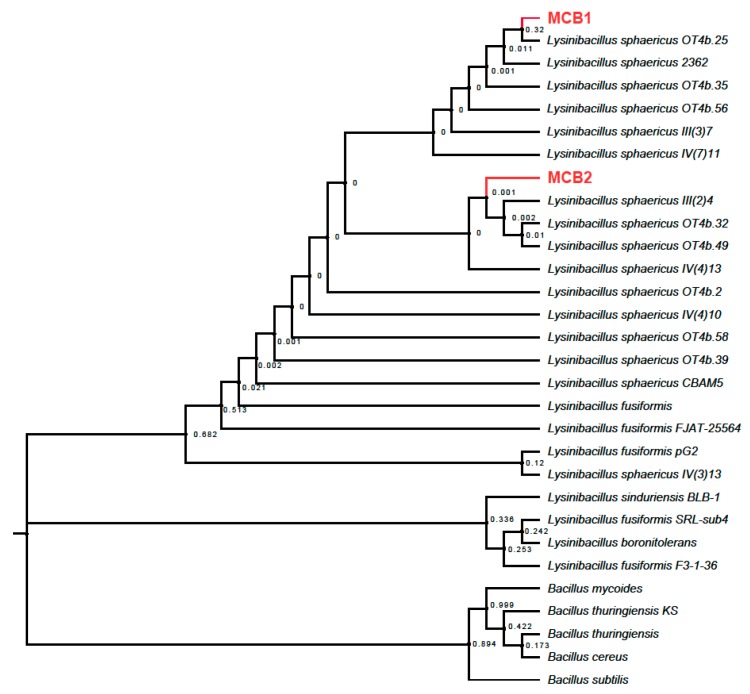
Phylogenetic tree for *Lysinibacillus sphaericus* with the two new strains isolated, MCB1 and MCB2.

A total of 12 morphotypes were identified as sporulated microorganisms. Although the majority of them were gram variable or positive bacilli, gram-negative cocci and bacilli were also found in the samples. Only two morphotypes were identified as *L. sphaericus* through PCR of complete 16S and a posterior sequencing and analysis in NCBI and RDP databases. The phylogenetic tree was inferred by using the Maximum Likelihood method based on the Kimura 2-parameter model [42]. The tree with the highest log likelihood is shown (Figure 1). The percentage of replicate trees, in which the associated taxa clustered together in the bootstrap test (1000 replicates), are shown next to the branches. Initial tree(s) for the heuristic search were obtained automatically by applying Neighbor-Join and BioNJ algorithms to a matrix of pairwise distances estimated using the Maximum Composite Likelihood (MCL) approach, and then the topology with superior log likelihood value was selected. A discrete Gamma distribution was used to model evolutionary rate differences among sites (5 categories (+G, parameter = 0.0500)). Evolutionary analyses were conducted in MEGA X [43].

Additionally, tests were also carried out as confirmation probes. In both cases, the shape of the spore was terminal, the toxic bioassays show mortality for the larvae of *C. quinquefasciatus* and *A. aegypti* and the presence of the S-layer protein was evident (Figure S1, Table S1). This protein is intrinsically associated with metal accumulation due to its characteristic morphology. It is the outermost layer of the microorganisms, composed of a single protein or a group of glycoprotein monomers and with a capability for self-assembly [44,45]. By virtue of this, microorganisms with S-layer are a potential tool for processes of bioremediation and biotechnology. Following these tests, the morphotypes were identified as *L. sphaericus*. Because they are new strains, their denotation in this study from now on will be MCB1 and MCB2 with the accession numbers MH447519 and MH447518, respectively, in the NCBI.

### 3.2. Metal Sorption in Living Cells of L. sphaericus Strains

**Figure 2:**
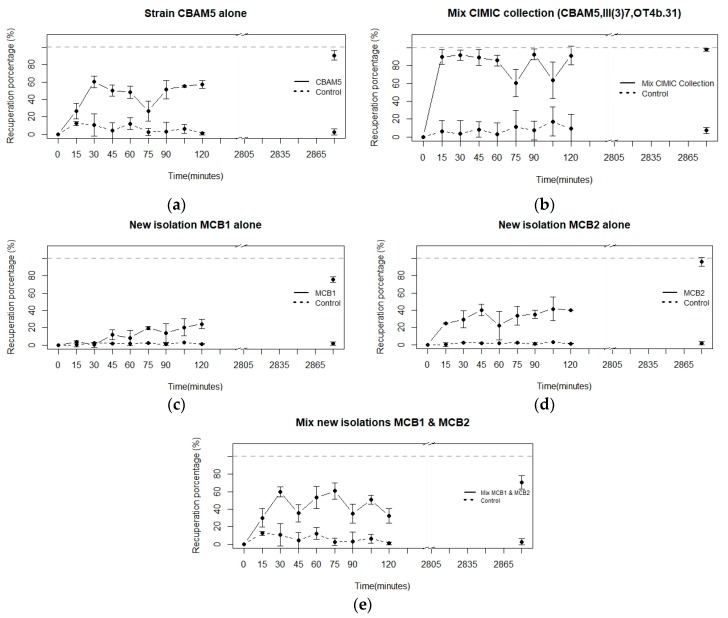
Bioassay in living cells with the *L. sphaericus* (**a**) CBAM5 as an individual strain, (**b**) CIMIC collection (CBAM5, OT4b.31, III(3)7) in a mix, (**c**) MCB1 and (**d**) MCB2 alone, and (**e**) mix with MCB1 and MCB2. The concentration of HAuCl_4_^−^ evaluated was 60 µg/mL in minimum salt medium. Graphs show deviation standard for each time evaluated.

The individual strain CBAM5, used as a calibration method, reached a percentage of recovery of 60% in the first 3 h of the bioassay and approximately 95% after 48 h (Figure 2a). The mixture of strains CBAM5, OT4B.31, and III(3)7 from the *L. sphaericus* CIMIC collection was the bioassay with the highest percentage of metal recovery, followed by CBAM5 alone (Figure 2a,b). Furthermore, CBAM5 had a similar percentage of metal recovery when is compared to the mixture between the new isolations of *L. sphaericus* MCB1 and MCB2, but this was reached in a shorter time, 30 min (Figure 2b,c). On the other hand, the highest percentage of metal recovery was 24.27% for MCB1 and almost the double for MCB2 at 41.45% (Figure 2c,d). A mixture of MCB1 and MCB2 also demonstrated the microorganism’s capability to capture this metal (Figure 2e). It is important to mention that the highest percentage of metal recovery (60.76%) was achieved in a shorter time when the strains were mixed than when they were used on their own (Figure 2c–e). Controls in all cases were above the absorbance for the treatments with *L. sphaericus* presence.

For all the bioassays performed, a similar pattern was detected. In the initial phase, a quick increment in the percentage of gold recovery was represented in a drastic fall of the absorbance. Subsequently, there were a series of oscillations until the end of the experiment (Figure 2).

As mentioned earlier, the presence of oscillations in the last stages of the experiments can suggest the presence of efflux pumps. Behavior models of these pumps were similar to the previously described reports for the same species but for the heavy metals, chrome and lead [21,39].

### 3.3. Biofabrication of Gold Nanoparticles (AuNPs)

**Figure 3:**
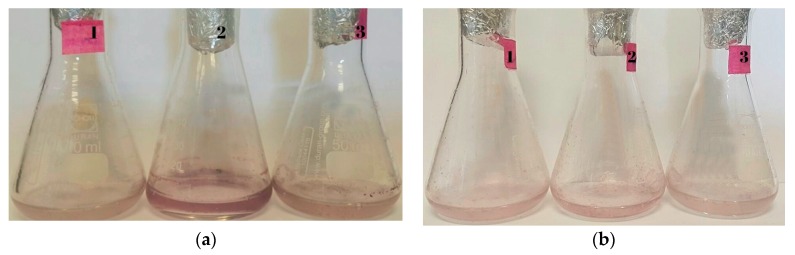

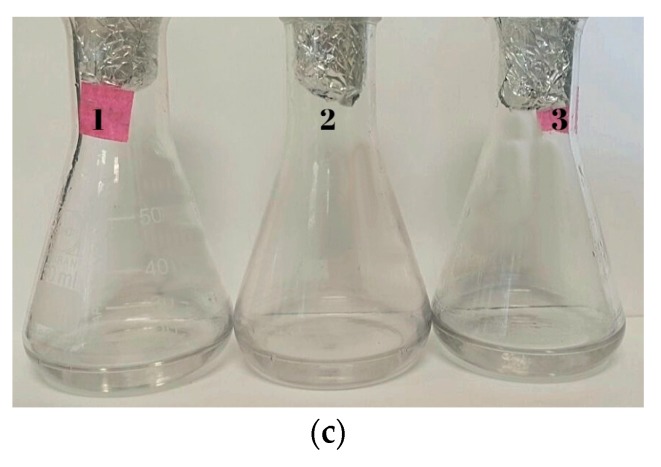
Bioassays of possible formation of gold nanoparticles with living cells for the strains (**a**) CBAM5 alone, (**b**) mix of MCB1 and MCB2 and, (**c**) control after 48 h. The numbers in the flasks are the replicates for each bioassay.

Possible biosynthesis of AuNPs in the strains evaluated was observed. After 48 h, the treatment flasks with the bacteria turned to a strong red-purple color (Figure 3). In the biological formation of AuNPs this color is very characteristic and in the majority of cases, it is a process that takes place several hours after the contact between the microorganism and the metal [28,29,46,47].

**Figure 4:**
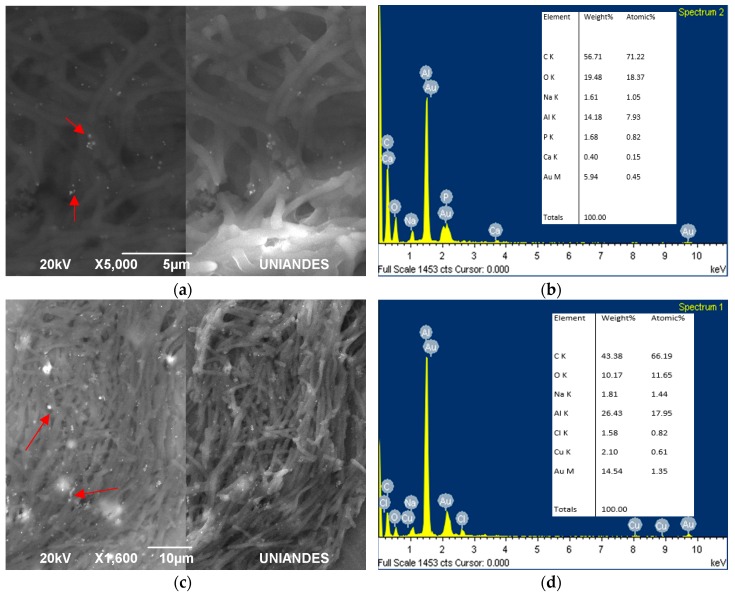

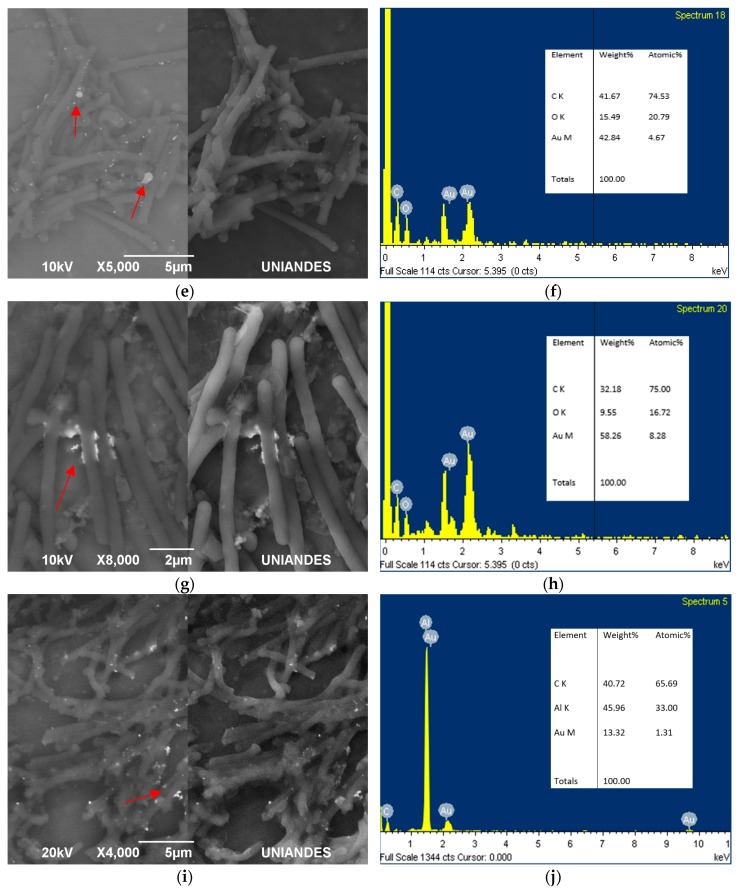
SEM images with SEI (secondary electrons) and BEC (Backscattered electrons) for bioassays with living cells of *Lysinibacillus sphaericus* (**a**) CBAM5 and (**b**) energy-dispersive X-ray spectroscopy (EDS) analysis; (**c**) mix of CIMIC collection strains (CBAM5, OT4b.31, III(3)7) with (**d**) EDS analysis. Additionally, *L. sphaericus* (**e**) strain MCB1 alone with its respective (**f**) EDS analysis and (**g**) MCB2 also with (**h**) EDS analysis. Similarly, the (**i**) mix between MCB1 and MCB2 and (**j**) EDS analysis.

For living cells, gold colloids did not have a specific formation around the cell or a clear pattern of accumulation; they were all over the surface (Figure 4). Additionally, some of the particles were not attached to the cells, but free in the area. These possible AuNPs varied in size from 50 nm to 100 nm. In general, the shape of all *L. sphaericus* strains were elongated and thin, possibly due to the pressure they were subjected to, with an acid pH solution and high gold concentrations. *L. sphaericus* CBAM5 shows clusters of possible, small AuNPs (Figure 4a,b). The possible AuNPs observed in the mixture between the strains from the CIMIC collection were not clumped, but rather dispersed throughout the surface covered by the cells (Figure 4c,d). MCB1 and MCB2 show bigger clumps of gold compared with the strains from the CIMIC collection (Figure 4e–h). Finally, the mixture of these strains also reveals dispersion all over the cell surface as in the mixture of the CBAM, OT4b.31, and III(3)7 strains (Figure 4i,j).

**Figure 5:**
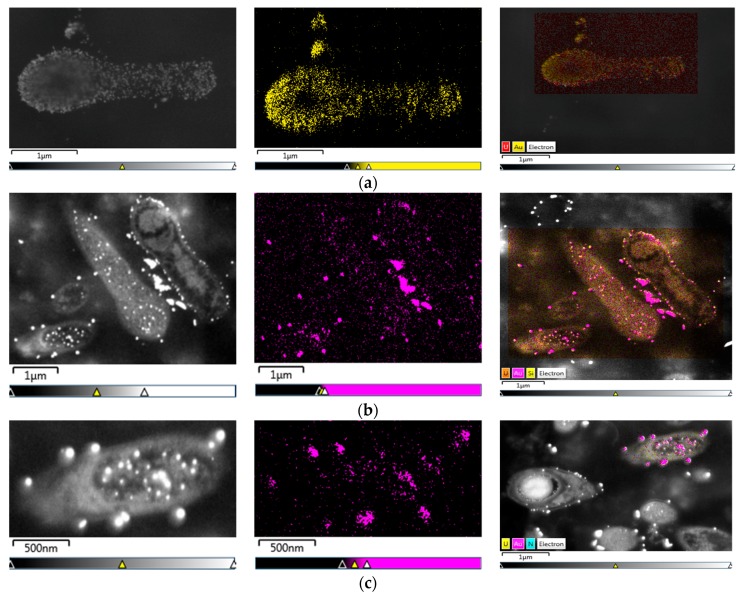
SEM and EDS analysis maps for samples of the mix between *Lysinibacillus sphaericus* MCB1 and MCB2 prepared in resin. Slides of 70 nm show cells (**a**,**b**) in different sporulation stages and in (**c**) a vegetative cell.

On the other hand, it was evident for the samples immersed in resin, that the gold was also inside the cell (Figure 5). The *L. sphaericus* strains MCB1 and MCB2 evaluated were able to adsorb and absorb gold, showing possible AuNPs inside and outside of the membrane. It was also remarkable that gold was also accumulated even when the cells were in the sporulation process (Figure 5a,b) or in vegetative stages (Figure 5c). The previous statement, suggests that the cycle stage of the microorganism is not an important factor that influences the biosynthesis or accumulation of the metal.

## 4. Discussion

The capability to bioabsorb gold of the two new strains of *L. sphaericus* MCB1 and MCB2 is indisputable, as well as of the strains of CIMIC collection. These abilities present great potential in the gold mining industry, particularly in the extraction process. Biological methodologies are generally low-cost techniques with high efficiencies, which make them attractive and adaptable to developing countries such as Colombia, where illegal and artisanal mining are present. These types of gold mining are characterized by the excessive use of mercury and cyanide, and the release of these heavy metals into the environment without any kind of pollution or health control [2,3]. The high efficiency shown by the strains, especially when in a mixture, makes *L. sphaericus* a promising candidate for gold biomining.

Bioassays using the mixtures had higher efficiencies than the ones with only one strain, displaying the benefits of mutualistic behavior by the microorganisms. It is also important to highlight the almost 100% recovery of gold by *L. sphaericus* MCB2, a native strain of the Nechi river at El Bagre, Colombia. This recovery was obtained in less than 50 h and with possible formation of gold nanoparticles or colloids.

Oscillations in the last stages of bioassays with live biomass suggest the presence of efflux pumps, an indirect mechanism of the cell for the introduction of divalent ions such as lead and chrome [21,40]. Also, this mechanism is proposed given that this metal does not bring any biological benefit to the cell. This strategy is known for other microorganisms because it is an evasion mechanism against metal accumulation and, consequently, it can confer resistance [48,49]. Additionally, *L. sphaericus* has a layer that surrounds the entire cell, known as the S-layer protein. The presence of the protein can confer important advantages to the microorganisms. It is demonstrated that bacteria with this protein possess diverse mechanisms for metal interactions and biotransformation; this includes biosorption and accumulation, but also acts as a protective structure in environments that are harmful for the cells such as active mining zones [50,51].

The possible synthesis of gold nanoparticles has many benefits for medicine, agriculture, the cosmetic industry, drug delivery, and biochemical sensors [52,53]. For example, AuNPs synthesized by the fungi *Candida albicans* have been studied as a tool for the early detection of liver cancer [54]. The general mechanism for microbial synthesis of gold nanoparticles is the reduction of Au (III) ionic form to Au(0) or AuNPs [26]. The mechanism involves electrostatic interactions of the positive charge that the metal ions have, with the negative charge of the cell wall [52]. Many metabolic processes are proposed for the bio-mineralization of gold by the microbes such as the presence of organic phosphate compounds, ligninases, laccases, reductases (NADH/NADPH), amino acids (tyrosine, cysteine and tryptophan), the lack of specific metal transport systems, and extracellular complexation and efflux systems [51,52]. The production of AuNPs can be associated to detoxification in toxic environments, transforming metal ions to insoluble complexes. In this sense, the ability to form metal agglomerations can be used as an eco-friendly technique in the mining industry for different metals.

We propose that the mechanism by which *L. sphaericus* can synthesize gold is the presence of *mer* clusters. It is known that these genes confer Hg resistance, and are inducible in the presence of low concentrations of heavy metals in the environment. It suggests a cross-regulation of the Hg systems by Au(III) because of their physicochemical similarity [26]. The specific mechanism by which this occurs in *L. sphaericus* is still unknown and it will be the aim for future research.

To continue research in this field, it is important to evaluate the selectivity of the strains for the metal, due to the fact that the water effluent may contain other heavy metals such as mercury. Additionally, studies estimating the potential of the S-layer protein should also be performed, given its capacity of accumulate metal ions. Finally, the development of a bioreactor as a pilot could be an important approach to the real conditions that *L. sphaericus* is subject to in obtaining gold without mercury or cyanide.

## 5. Conclusions

The two new strains of *L. sphaericus*, as well as the ones from the CIMIC collection, show an elevated percentage of gold recovery, reaching 95% in less than 50 h. This bacterium can be a potential tool in the extraction of gold in Colombia due to its diverse advantages including efficiency, fast processing, the low cost of the technique, an environmentally friendly approach, health safety, and the use of native strains. These strains also have the ability to bio-produce possible gold nanoparticles in short time intervals, making them a more attractive alternative.

## Figures and Tables

**Table 1:** Strains evaluated in the bioassays of this study.

*Lysinibacillus sphaericus* Strains	Site of Isolation	Reference
MCB1	Nechi River	This study
MCB2	Nechi River	This study
CBAM5	Hydrocarbon-contaminated soil	[30]
III(3)7	Oak forest soil	[30]
OT4b.31	Coleopteran larvae	[30]

## Supplementary Materials

The following are available online at http://www.mdpi.com/1996-1944/11/9/1587/s1, Figure S1: Image of optic microscope 100× and SEM of sporulation cycle of *L. sphaericus* MCB1 and MCB2. Figure S2: SDS-PAGE gel of S-layer purification in MCB1 and MCB2, Table S1: Mortality bioassays with *L. sphaericus* on larvae of *C. quinquefasciatus* and *A. aegpti*.
