# Supplementary material for: The Golden Activity of Lysinibacillus sphaericus: New Insights on Gold Accumulation and Possible Nanoparticles Biosynthesis
Source: Materials (Basel). 2018 Sep 2;11(9):1587. doi: 10.3390/ma11091587 (PMC6163967; doi:10.3390/ma11091587)
Supplement: Supplementary file 1 [file materials-11-01587-s001.pdf]

Supplementary Materials:

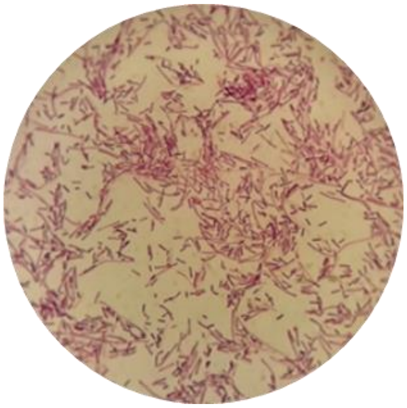

(a)

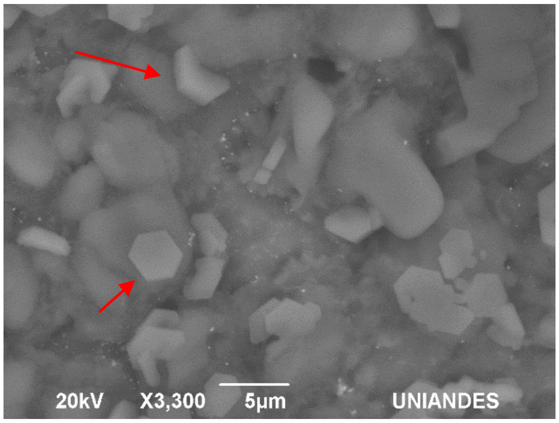

(b)

Figure S1: Image of optic microscope 100x of (a) strain MCB2 showing different stages of sporulation cycle. Additionally, (b) SEM observation of crystal protein during sporulation of the mix between *L. sphaericus* MCB1 and MCB2.

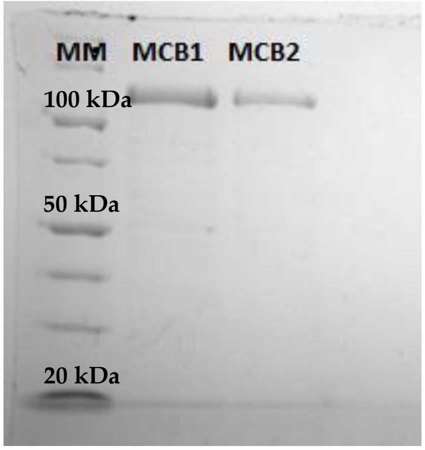

Figure S2: SDS-PAGE gel of S-layer purification in MCB1 and MCB2

Table S1: Mortality bioassays with *L. sphaericus* on larvae of *Culex quinquefasciatus* and *Aedes aegypti*. Experiments were carried out in triplicate and the control was only with the larvae, while *L. sphaericus* strain 2362 was use as positive control.

	24 hours		48 hours	
**Treatment**	*C. quinquefasciatus*	*A. aegyti*	*C. quinquefasciatus*	*A. aegyti*
Control	0 ± 0,000	1 ± 1,527	5 ± 1,000	1 ± 1,000
2362	18 ± 0,577	1 ± 0,577	18 ± 0,000	5 ± 1,154
MCB1	18 ± 1,732	5 ± 1,732	19 ± 1,154	7 ± 1,527
MCB2	1 ± 0,577	1 ± 0,577	16 ± 0,577	3 ± 2,309
